# THE NATIONAL STRATEGY TO SUPPORT FAMILY CAREGIVERS: A STATE-LEVEL PERSPECTIVE ON IMPLEMENTATION

**DOI:** 10.1093/geroni/igad104.1644

**Published:** 2023-12-21

**Authors:** Salom Teshale

**Affiliations:** The National Academy for State Health Policy, Washington, District of Columbia, United States

## Abstract

The 2022 National Strategy to Support Family Caregivers includes action steps that a wide range of non-federal organizations, including states, can take to support family caregivers. States have a variety of ways that they can approach supporting family caregiver policy via action steps, including but not limited to strengthening access to services, collaborating among agencies and organizations to coordinate activities, and tracking caregiver data to help ensure services are meeting caregiver needs. Several states are taking the lead in promoting use of caregiver assessment tools. States can monitor whether initiatives and activities are achieving their goals of supporting caregivers and care recipients through tracking the implementation of the state’s actions to support family caregivers. Drawing on the National Academy for State Health Policy’s role in supporting states through its Resource and Dissemination Center, this presentation will highlight ranges of action steps in the National Strategy that states can take to support family caregivers; note frameworks that states can consider using to track implementation of action steps; and highlight states’ initiatives aligning with action steps.

